# Bonding-restricted structure search for novel 2D materials with dispersed C_2_ dimers

**DOI:** 10.1038/srep29531

**Published:** 2016-07-12

**Authors:** Cunzhi Zhang, Shunhong Zhang, Qian Wang

**Affiliations:** 1 Department of Materials Science and Engineering, College of Engineering, Peking University, Beijing 100871, China; 2 Center for Applied Physics and Technology, Peking University, IFSA Collaborative Innovation Center, Key Laboratory of High Energy Density Physics Simulation, Ministry of Education, Beijing 100871, China

## Abstract

Currently, the available algorithms for unbiased structure searches are primarily atom-based, where atoms are manipulated as the elementary units, and energy is used as the target function without any restrictions on the bonding of atoms. In fact, in many cases such as nanostructure-assembled materials, the structural units are nanoclusters. We report a study of a bonding-restricted structure search method based on the particle swarm optimization (PSO) for finding the stable structures of two-dimensional (2D) materials containing dispersed C_2_ dimers rather than individual C atoms. The C_2_ dimer can be considered as a prototype of nanoclusters. Taking Si-C, B-C and Ti-C systems as test cases, our method combined with density functional theory and phonon calculations uncover new ground state geometrical structures for SiC_2_, Si_2_C_2_, BC_2_, B_2_C_2_, TiC_2_, and Ti_2_C_2_ sheets and their low-lying energy allotropes, as well as their electronic structures. Equally important, this method can be applied to other complex systems even containing *f* elements and other molecular dimers such as S_2_, N_2_, B_2_ and Si_2_, where the complex orbital orientations require extensive search for finding the optimal orientations to maximize the bonding with the dimers, predicting new 2D materials beyond MXenes (a family of transition metal carbides or nitrides) and dichalcogenide monolayers.

Determining the geometrical structures is essential to design new materials. The traditional X-ray diffraction technique for determining the crystal structure requires high quality samples, and is incapable of dealing with materials under extreme conditions such as high pressure, strong electric or magnetic fields. On the other hand, determining a ground-state geometry based on chemical and physical intuitions is difficult due to the complex potential energy surface especially for materials with large unit cells or complicated structural units. Hence many structure prediction methods based on quantum mechanical simulations combined with global optimization algorithms have been developed, such as simulated annealing^1^, genetic algorithm^2^,^3^, and basin hopping^4^. However, in these currently available methods, energy is used as the target function and there are no restrictions on the bonding of atoms, and atoms are usually manipulated as the elementary units. Thus, they are unsuitable for doing structure search for nanocluster-assembled materials where the structural units are nanoclusters, which are not directly bonded to each other but through linkers in most cases. Therefore, it is highly desirable to develop new search algorithms for doing such bonding-restricted structure search. An earlier work reported a constrained evolutionary search algorithm that enables prediction of polymeric crystal^5^. However, algorithms for motif-based prediction of low dimensional materials are not yet reported.

In this study, we report a dimer-based bonding-restricted search method for finding stable 2D structures with a restriction of no direct bonding between the dimers. C_2_ is a well-known pseudohalogen unit with an electron affinity of 3.4 eV^6^, and is the basic structural unit of diverse carbon compounds. It has received increasing attention due to its rich physical and chemical properties. R. Hoffmann^7^ recently made a comment on C_2_: “C_2_ is one of my favorite molecules, for this diatomic epitomizes many of the fundamental questions of chemistry”. Shaik and co-workers^8^ demonstrated that an unusual quadruple bond may exist in C_2_, which is strong enough to be considered as a typical chemical bond. In addition, dimerization of carbon is not only the first stage toward final crystallization, but also plays a vital role in the growth of many carbon related materials^9^,^10^. For instance, a C_2_ dimer on a Cu surface was identified as the dominant feeding species for graphene growth due to its lower diffusion and attachment barrier as compared to the carbon monomer and trimer^11^,^12^. Although the C_2_ dimer is quite chemically active and inclined to aggregate, it naturally occurs in the carbon vapor of interstellar medium and electric arcs. Vapor from a 16-V carbon arc was found to contain 28 wt% diatomic carbon^13^. As a result, in molecular dynamics (MD) simulations of growth of carbon–based materials, such as graphene, carbon nanotube and C_60_, C_2_ dimers are usually adopted as the initial carbon source^14^,^15^,^16^.

Furthermore, the C_2_ dimer is the building block of metal-alkynide complexes^17^, alkynide complexes^18^, metallocarbohedrenes clusters M_*m*_C_*n*_^19^,^20^ (usually termed as metcars) and ternary metal carbides (LiAgC_2_, KAgC_2_, CsAgC_2_ and NaPdC_2_)^21^. In recently studied silicon carbides (Si_x_C_y_), the dimerization of C in the SiC monolayer reintroduces the Dirac cone into the honeycomb lattice^22^, while SiC sheets without C_2_ dimer are wide-band gap semiconductors^23^,^24^,^25^,^26^. In SiC_2_ silagraphene^27^, each silicon atom binds to four C_2_ units in a flat plane, resulting in a metallic sheet. When C_2_ dimers are embedded in a 2D porphyrin sheet, the system becomes highly active for oxygen reduction reaction^28^. In addition, it was demonstrated that the formation of C_2_ dimers plays an important role in stabilizing metcar clusters^29^.

Due to the C_2_ dimer’s unique properties as well as its importance in forming numerous carbon-based three-dimensional materials and zero-dimensional nanostructures, we focus this study on developing a dimer-based global search algorithm to design 2D materials containing dispersed C_2_ dimers to retain its intrinsic properties. We then apply this method to identify the ground state geometries of the 2D Si-C_2_, B-C_2_, and Ti-C_2_ systems containing C_2_ dimers as test cases.

## Results and Discussion

To test our method, we applied it to three systems where C_2_ dimers are linked with Si, B and Ti atoms respectively. New ground state geometries and new low-lying isomers are identified.

### 2D Si-C_2_ system

Recently, SiC_2_ and Si_2_C_2_ sheets with dispersed carbon dimers have received some attention. The studies were carried out using *a priori* geometric structures to investigate the properties of 2D Si-C sheets^22^,^27^,^30^. However, a complete understanding of Si-C_2_ phases is still lacking, and it is unclear whether the studied structures are the ground states under the condition of discrete C_2_ dimers. Thus we have conducted a comprehensive structure search to identify the ground state geometry and the low-lying energy isomers of the Si-C_2_ system.

**Figure 1:**
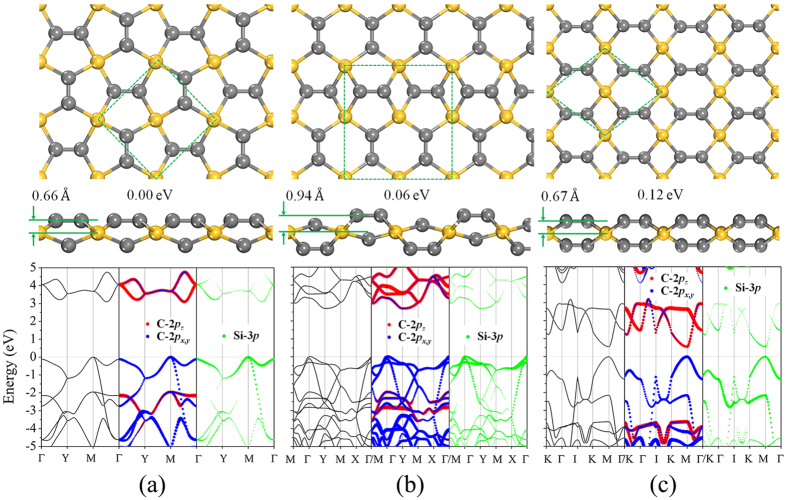
Geometrical and electronic structures. Top and side views of the atomic configurations of C_2_ dimer-based 2D SiC_2_ isomers and their corresponding electronic band structures. (**a**) penta-SiC_2_, (**b**) 456-SiC_2_, and (**c**) 46-SiC_2_. Grey and yellow spheres represent C and Si atoms, respectively. The relative energy/f.u. with respect to penta-SiC_2_ is given below each structure. Decomposed band structures (bottom panel) show the contribution of different orbitals.

The most stable structure of Si_2_C_2_ and three low-lying energy isomers of SiC_2_ have been identified using our C_2_ dimer-based global search. Because the lowest energy geometry of Si_2_C_2_ is found to be identical to a recently reported structure^22^, we only concentrate on the three isomers of the SiC_2_ sheet, as shown in Fig. 1. In these structures all carbon atoms are pairwisely bonded with each other and covalently bonded with Si atoms. They all are comprised of four-fold coordinated Si with three-fold coordinated C atoms. The structure, shown in Fig. 1(a), is composed of pure pentagons, thus it is named penta-SiC_2_. The other two isomers shown in Fig. 1(b,c) consist of 4-, 5- and 6-rings, and 4- and 6-rings, respectively, labeled as 456-SiC_2_ and 46-SiC_2_ accordingly. Si-C and C-C bond lengths are about 1.90 Å and 1.36 Å respectively. The side views show the sandwich-like structures with the four-fold coordinated Si atoms in the middle sandwiched between C_2_ dimers. When we consider the Si sublattice and C_2_ dimer (treating C_2_ as a structural unit) sublattice separately, it turned out that the major difference among these three isomers is the orientation of C_2_ dimers, namely the dimers are parallel to each other in 46-SiC_2_, while they are perpendicular to each other in penta-SiC_2_ (In Supplementary Information, we shortly discuss the kinetic barriers connecting different isomers). It is obvious that the different arrangement of C_2_ dimers remarkably alters the geometrical structures, resulting in the different electronic properties of these isomers as demonstrated in the following paragraph.

Total energy calculations reveal that all three isomers are energetically more favorable than the previously proposed SiC_2_-silagraphene^27^ with planar tetracoordinate silicon atoms. Penta-SiC_2_, 456-SiC_2_ and 46-SiC_2_ are 0.63, 0.57, and 0.51 eV/formula unit (f.u.) lower in energy than that of SiC_2_-silagraphene, suggesting that the Si-C_2_ sheet is more likely to adopt a buckled structure with the partially *sp*^3^ hybridized Si and the *sp*^2^ hybridized C. Penta-SiC_2_ is found to be the lowest energy configuration among the three isomers with the Si-C and C-C bond lengths of 1.90 Å and 1.36 Å respectively, which is identical to that reported recently by Bezanilla *et al*.^30^, and as 456-SiC_2_ lies only 0.06 eV higher in energy than penta-SiC_2_, the two structures can be considered as energetically degenerate. The relative energy of 46-SiC_2_ with respect to penta-SiC_2_ is 0.12 eV. The dynamical stability of the predicted low-lying energy isomers of SiC_2_ is verified by calculating their phonon dispersions. The results plotted in Fig. S1 (see Supplementary Information) show that the three structures are all dynamically stable since their vibration modes are real in their entire Brillouin zones, respectively.

To understand the electronic properties of the three isomers, their band structures are calculated using the HSE06 hybrid functional. Furthermore, a more detailed investigation of the contribution from each atomic orbital to the band structure is carried out using the color-mapped bands. The calculated results are presented in Fig. 1, which shows that penta-SiC_2_, 456-SiC_2_ are large band gap compounds with band gaps of 3.1 eV, 2.7 eV, while the bandgap of 46-SiC_2_ is 0.55 eV. We note that 46-SiC_2_ is a direct band gap semiconductor and its band gap is much smaller than that of penta-SiC_2_ and 456-SiC_2_. While the others are indirect band gap semiconductors because the valence band maximum (VBM) and the conduction band minimum (CBM) are not at the same point in their Brillouin Zones. The localized distribution of *p*_*z*_ orbitals of the C_2_ dimer, above and below the Fermi level, is disclosed in the colored band structure (red dot curves in decomposed band structure). Further analyses of the chemical bonding of the C_2_ dimer reveals that the localized distribution originates from the bonding and anti-bonding *p*_*z*_ orbital of C_2_ dimers. Due to the unique geometrical structure, the alternation of partially *sp*^3^ hybridized Si and slightly distorted *sp*^2^ C prevents the *p*_*z*_ orbitals from forming a delocalized π bond. This could explain, to some degree, why these *p*_*z*_ composed bands are less dispersive than those in graphene or other planar silicon carbon sheets.

To understand the origin of the different electronic structures exhibited in the three isomers, the detailed structural configurations of the local C and Si atoms in penta-SiC_2_ and 46-SiC_2_ are sketched in Fig. S3 (see Supplementary Information). Penta-SiC_2_ has higher symmetry subjecting it to the least geometrical distortion among the three isomers. 46-SiC_2_’s parallel arrangement of the C_2_ dimer imposes a larger geometrical distortion; the angle of C with its neighboring Si is 92.9 °, while that in penta-SiC_2_ is 109.6 °. The Si atoms in 46-SiC_2_ also adopt the distorted hybridization as compared to that in penta-SiC_2_. Besides, the decomposed band structures (Fig. 1) show that the highest and the second highest occupied bands near the M point in 46-SiC_2_ are exclusively from the Si-C *σ* bonds. The larger geometrical distortion of Si-C *σ* bonds in 46-SiC_2_ leads to a stronger interaction between the relevant orbitals, resulting in the highly dispersive bands and a smaller band gap.

### 2D B-C_2_ system

For the second test system, we study the 2D B-C_2_ system due to boron’s rich chemistry: for example its electron deficiency and versatile bonding ability. In fact, nanostructures composed of boron and carbon atoms have attracted growing interest both theoretically and experimentally. For instance, a BC_3_ honeycomb structure with high crystalline quality was identified by electron diffraction^31^. A B_2_C sheet with planar tetra-coordinated carbon (pt-C) moiety was theoretically predicted^32^. A thorough study of B-C compounds with different stoichiometry was carried out by performing a PSO search^33^. However, a study on B-C sheets comprised of discrete C_2_ dimers has not been reported. Due to its versatility in forming various chemical bonds and the differing chemical nature of B from Si, the bonding between B and the C_2_ dimer is expected to be quite different from that of Si and C_2_, leading to B-C_2_ systems with different structural and electronic properties.

**Figure 2:**
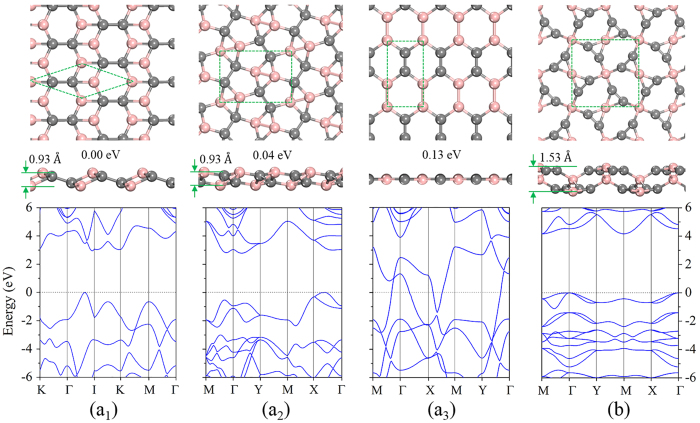
Geometrical and electronic structures. Top and side views of the atomic configurations and their band structures of B-C_2_ (**a**_**1**_) B_2_C_2_-a, (**a**_**2**_) B_2_C_2_-b, and (**a**_**3**_) B_2_C_2_-c, and (**b**) BC_2_.

Using our dimer-based search algorithm, three low-lying energy isomers of B_2_C_2_ and one stable structure of BC_2_ are obtained (Fig. 2). Geometry optimizations and total energy calculations suggest that the structure shown in Fig. 2(a1) has the lowest energy of the three isomers of B_2_C_2_ with a total energy of 0.04 eV, 0.13 eV lower than that of the structures shown in Fig. 2(a2,a3), respectively. To examine the dynamical stability of these structures, we calculated their phonon dispersions. The results are plotted in Fig. S4. The absence of imaginary modes in the entire Brillouin zone for each of the structures confirms that they are all dynamically stable. However, we note that these structures are energetically metastable as compared to the B-C planar monolayers predicted by Luo *et al*. (B_2_C_2_-a is 0.23 eV/f.u. higher in energy than the most stable BC sheet).

**Figure 3:**
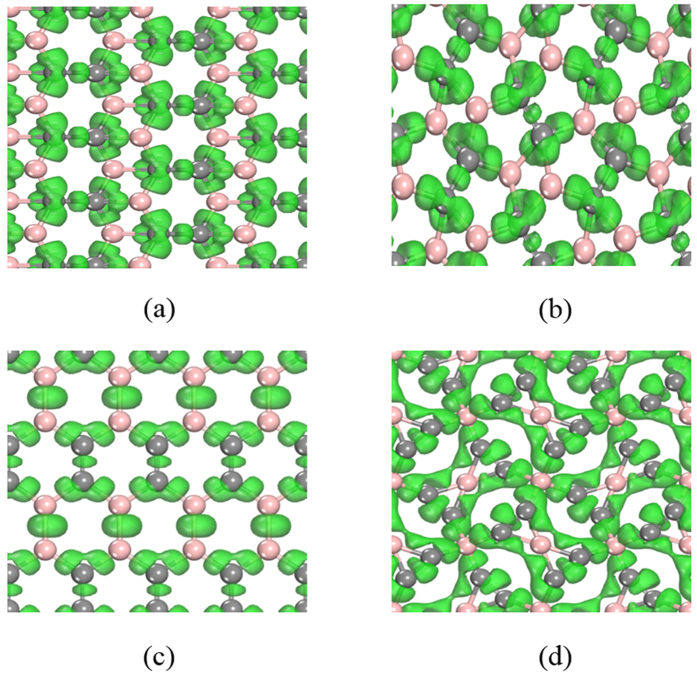
Electron localization function Isosurface. Isosurfaces of electron localization function (ELF) with the value of 0.75 for the structures (**a**) B_2_C_2_-a, (**b**) B_2_C_2_-b, (**c**) B_2_C_2_-c and (**d**) BC_2_.

In order to gain more insight into the chemical bonding in the B-C_2_ sheets, we plotted an electron localization function (ELF)^34^ isosurface (with a relatively large isovalue of 0.75 to reflect the σ bonds between the atoms) in Fig. 3. According to the ELF diagrams, carbon in B_2_C_2_-a and B_2_C_2_-b adopt *sp*^3^ hybridization, and each carbon atom is covalently bonded to three adjacent B and one C atoms. B atoms take a slightly distorted *sp*^2^ configuration. The C-C bond length in Fig. 2(a1,2) are 1.51 Å (close to that of a C-C single bond). While in B_2_C_2_-c, both the B and C atoms have *sp*^2^ hybridization. B_2_C_2_-c adopts a planar honeycomb structure similar to graphene^35^, however, the total energy of B_2_C_2_-c is 0.13 eV/f.u. higher than that of B_2_C_2_-a. Multi-center bonds are pretty common in boron-related materials^36^,^37^, and contribute a lot to their stability. However, no such bonds are found in B_2_C_2_-a, B_2_C_2_-b and B_2_C_2_-c, which might explain the physical origin of their higher energy.

For the BC_2_ sheet, only one stable structure without imaginary modes was discovered (Figs 2(b) and S4(b)). In particular, all the BC_2_ structures isostructural to those of SiC_2_ (namely penta-BC_2_ or 46-BC_2_) are unstable, reflecting the differing chemical nature of B and Si. In this highly buckled BC_2_ sheet (the thickness is 1.53 Å), all C atoms are strongly covalently bonded together; the C-C bond length is 1.31 Å. The calculated electron localization function (ELF) shows that there are *sp*^3^ hybridized B and nearly planar tetracoordinate B atoms. In our case, the bonding type of planar tetracoordinated B is closer to the case of planar tetracoordinated Si^27^ and C^32^.

Our electronic band structure calculations (Fig. 2) show that the ground state structures of B_2_C_2_ and BC_2_ with the discrete C_2_ dimers have indirect band gaps of 2.9, 2.8 and 4.1 eV at the HSE06 level for B_2_C_2_-a, B_2_C_2_-b and BC_2_, respectively, which is in contrast to the metallic BC sheet^33^ where C is not in C_2_ form. The reason is, once we restrict the carbon atoms to be pairwisely bonded together, the most stable structures (B_2_C_2_-a and BC_2_) discovered by using our dimer-based search are buckled. The separation of C_2_ dimers by boron atoms and the buckling nature of the sheets would destroy the delocalization of *p*_z_ π electrons of boron and carbon (the main reason for the metallicity of many planar stable B-C compounds). In the B_2_C_2_-c isomer, the planar geometry, as well as its delocalized π electrons is preserved. Geometrically speaking, B_2_C_2_-c is similar to honeycomb SiC^22^. Since boron has few valence electrons it is impossible to fill all the electronic states below the Dirac cone giving rise to metallicity.

### 2D Ti-C_2_ system

To study the interaction between the C_2_ dimer and transition metal elements, we have systematically studied Ti-C_2_ sheets for the following reasons: (1) both Ti and Si possess four valence electrons, thus it is intriguing to look at the difference between TiC_2_ and SiC_2_ sheets and Ti_2_C_2_ and Si_2_C_2_ sheets. (2) Titanium and carbon have been found to form various kinds of stable compounds ranging from 0D clusters^19^,^38^, to 2D sheets^39^,^40^, and to 3D bulk materials. Among them, in metcar clusters all carbon atoms exist in the form of a C_2_ dimer^19^. Therefore, it might be possible to find a family of 2D crystals that consists of transition metal atoms and C_2_ dimers. (3) Recently, a group of 2D layered early transition metal carbides termed as MXenes, including Ti_3_C_2_ and Ti_2_C, were experimentally synthesized by exfoliation from their bulk MAX phases^41^,^42^,^43^. However, in MXenes sheets, C is in atomic form rather than a C_2_ dimer. Therefore it is necessary to study the possibility of forming stable 2D titanium carbides containing C atoms that are all in C_2_ dimer form.

**Figure 4:**
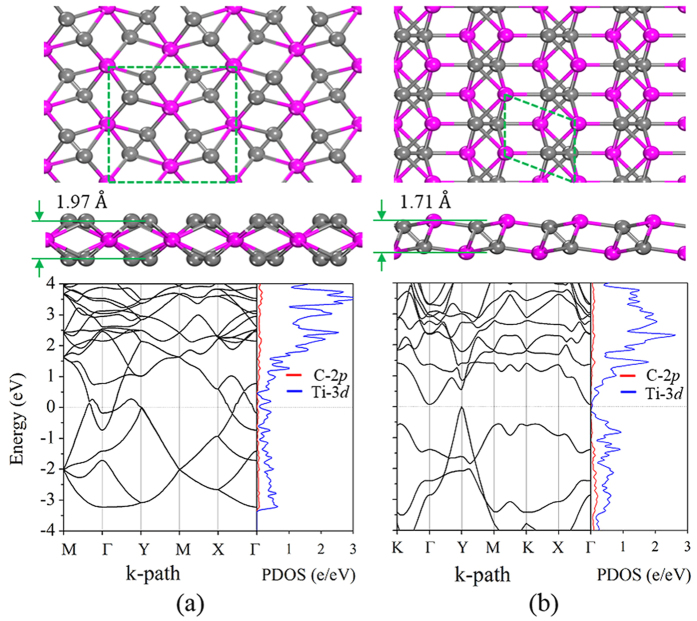
Geometrical and electronic structures. Top and side views of the crystal structures of (**a**) TiC_2,_ and (**b**) Ti_2_C_2_, and their corresponding electronic band structures and partial DOS. Magenta and grey spheres represent Ti and C atoms, respectively. Their dynamical stabilities are confirmed by phonon calculations (see Fig. S5 in Supplementary Information).

We concentrated on the 2D titanium carbide sheets with the stoichiometry of TiC_2_ and Ti_2_C_2_ where C is in the form of a C_2_ dimer. Recently, a TiC_2_ sheet was predicted to have good performance as anode material for a lithium ion battery^40^. However, the geometrical structure was artificially designed. Thus, it is easy to miss some low-lying isomers. In fact, we found, using our dimer-based algorithm, that among the isomers of TiC_2_, there is a one that is lower in energy by 0.13 eV/f.u. than the recently reported structure^40^. In this lowest energy structure, as shown in Fig. 4(a), due to the different orientations of C_2_ dimers, the total thickness of 1.97 Å is less than the 2.02 Å of the reported structure^40^. One can see when going from Si to Ti, although both have four valence electrons, the complex 3*d* orbitals in Ti require a delicate arrangement of C_2_ dimers to match with the *d* orbital orientations for maximizing the interactions. The stability of the TiC_2_ sheet can be traced back to the most stable 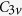-like Ti_8_C_12_ cluster^44^, where all carbon dimers bind to Ti in end-on configuration (EOC) and side-on configuration (SOC), which strengthens the bonding between Ti and C and stabilizes the Ti_8_C_12_ cluster. In our 2D TiC_2_ sheet EOC and SOC are apparently well preserved. The C-C bond length is 1.33 Å, which is slightly longer than that of a dispersed C_2_ molecule (1.31 Å) due to the partial occupation of π_g_ orbital of C_2_ in TiC_2_. The bond length of Ti-C in EOC and SOC modes is 2.05 Å and 2.19 Å, respectively, close to 2.1 Å in Ti_2_C MXene phase.

We then increased the atomic ratio of Ti from TiC_2_ to Ti_2_C_2_ to study the effect of Ti concentration on the geometry and properties of the Ti-C sheets. The structure shown in Fig. 4(b) is found to have the lowest energy among the isomers of Ti_2_C_2_ under the requirement of discrete C_2_ dimers, and it lies lower in energy by 0.25 eV/f.u than the t-TiC sheet^39^. This suggests that when the same atomic ratio of Ti to C exists, C_2_ is energetically more favorable than atomic C to bind with Ti in forming a sheet. The C-C bond length is 1.48 Å, close to that of C-C single bond but longer than that in a TiC_2_ sheet. This is because the hybridization between the C_2_-π_g_ and Ti-3*d* is stronger in Ti_2_C_2_, resulting in more π_g_ states being occupied, thus leading to the elongated C-C bond.

The calculated band structures and partial DOS are also plotted in Fig. 4, which clearly show that the TiC_2_ sheet is metallic, while the Ti_2_C_2_ sheet, like the t-TiC sheet, is an indirect narrow gap semiconductor with a gap of 0.1 eV at the HSE06 level. It is known that the ground state electronic configuration of an dispersed C_2_ is (1σ_g_)^2^(1σ_u_)^2^(2σ_g_)^2^(2σ_u_)^2^(1π_u_)^4^ with the higher energy states (3σ_g_)(1π_g_)(3σ_u_) unoccupied, and the (2σ_g_)^2^(2σ_u_)^2^(1π_u_)^4^(3σ_g_) having much lower energy as compared to the orbitals of Ti-3*d*. Therefore, a weak interaction between the deep level orbitals of C_2_ and Ti-3*d* is expected. Thus, the electronic states near the Fermi level are mainly from the interplay between C_2_-π_g_ and Ti-3*d* orbitals.

For the structure of t-TiC^39^ a strong electronic resonance between C-2*p* and Ti-3*d* is obvious. Due to the strong hybridization between C-2*p* and Ti-3*d* and the low buckled geometry of t-TiC, the bonding states of C-2*p* and Ti-3*d* are separated from the unoccupied Ti-3*d* states forming a band gap. TiC_2_ is geometrically analogous to t-TiC with carbon atoms replaced with discrete C_2_ dimers. However, replacing C with C_2_ leads to drastic change in their electronic structures, namely t-TiC is semiconducting with a strong electronic resonance, while TiC_2_ is metallic. This is because the π_g_ orbitals of C_2_ hybridize with the Ti-3*d* orbitals, forming the energy bands crossing the Fermi level, thus, resulting in metallicity of TiC_2_. For the Ti_2_C_2_ sheet, the electronic states near the Fermi level are also derived from C_2_ π_g_ and Ti 3*d* orbitals similar to TiC_2_, yet the interaction between C_2_ π_g_ and Ti 3*d* orbitals is stronger as compared to that in TiC_2_ due to the higher Ti concentration, which reduces the electronic states near the Fermi level, and thus results in the small band gap as shown in Fig. 4(b).

Based on the above discussions, the following intriguing points can be concluded: (1) compared with the sandwiched structure of the TiC_2_ sheet, the Ti atoms are more exposed in the Ti_2_C_2_ sheet, and hence have higher chemical reactivity; (2) Although having a higher atomic ratio of Ti, the Ti_2_C_2_ sheet is semiconducting with a band gap of 0.1 eV, while the TiC_2_ sheet is metallic; (3) For the t-TiC^39^ and Ti_2_C_2_ sheets, although they have the same composition ratio, when going from atomic C to C_2_ dimer, the band gap is reduced from 0.2 eV to 0.1 eV, showing promising applications of the Ti_2_C_2_ sheet in infrared light-related technology.

### Summary

In this study, we developed a dimer-based bonding-restricted structure search method to find the ground state structures of the 2D materials containing C_2_ dimers with a restriction of no direct bonded dimers, and then applied this method to three systems composed of discrete C_2_ dimers that are coordinated with Si, B and Ti atoms, respectively, as test cases. For the SiC_2_ sheet, three energetically nearly degenerate allotropes with very different electronic structures are identified, which also differ from the previously proposed SiC_2_-silagraphene^27^, showing that tuning the orientation of the C_2_ dimers not only can modulate the size of the band gap, but also can induce the transition from an indirect gap to a direct gap. For the B-C system, the ground state structures of both BC_2_ and B_2_C_2_ sheets are determined. Due to the different chemical nature of B and Si, all the possible structures of BC_2_ isostructural to those of SiC_2_ are unstable. In the ground state configuration of BC_2_, each C_2_ dimer is bonded with three B atoms forming a 2D sheet with a thickness is 1.53 Å and a band gap of 4.1 eV. When increasing the number of atomic B to form B_2_C_2_, each C_2_ dimer is bonded to six B atoms having the maximum number of B-C σ bonds and reducing the band gap to 2.9 eV. Unlike the metallic BC sheet^33^ where C atoms do not form any dimers, both the BC_2_ and B_2_C_2_ sheets identified here are semiconducting. The emergence of band gap is attributed to the buckled structures, which prevents the delocalization of *p*_*z*_ orbitals. For the Ti-C system, a new structure of the TiC_2_ sheet with a lower energy than the metallic TiC_2_ sheet reported recently^40^ was found. A Ti_2_C_2_ sheet was also found to be energetically more stable than the previously proposed t-TiC sheet^39^, suggesting it is more favorable for Ti to bind with C_2_ dimers in forming 2D structures. Unlike the metallic TiC_2_ monolayer^40^, the Ti_2_C_2_ sheet is an indirect band gap semiconductor. In addition, compared with the structures composed of C_2_ dimers and nonmetallic elements (Si, B), both the stable TiC_2_ and Ti_2_C_2_ sheets adopt relatively more compact structures due to the complex orientations of *d* orbitals, which require a more extensive search to find the optimal orientation of the C_2_ dimers. These cases show that our searching algorithm is efficient and indispensable to design new 2D materials beyond the atom-based ones.

## Methods

### Dimer-based search algorithm

Particle Swarm Optimization (PSO), one of the most popular swarm intelligence algorithms, was originally developed by Kennedy and Eberhart in 1995^45^. The PSO algorithm utilizes the collective intelligence of the whole population rather than a single individual, it is quite efficient in handling various optimization problems. Recently, the PSO algorithm has been successfully applied to crystal structure prediction^46^,^47^,^48^, another intractable optimization problem. Just like in the natural world where collective intelligence facilitates the locating of food or optimal habitats for insects and fishes, PSO implementation in CALYPSO was proven to be efficient in predicting the most stable structures for undiscovered materials^48^.

**Figure 5:**
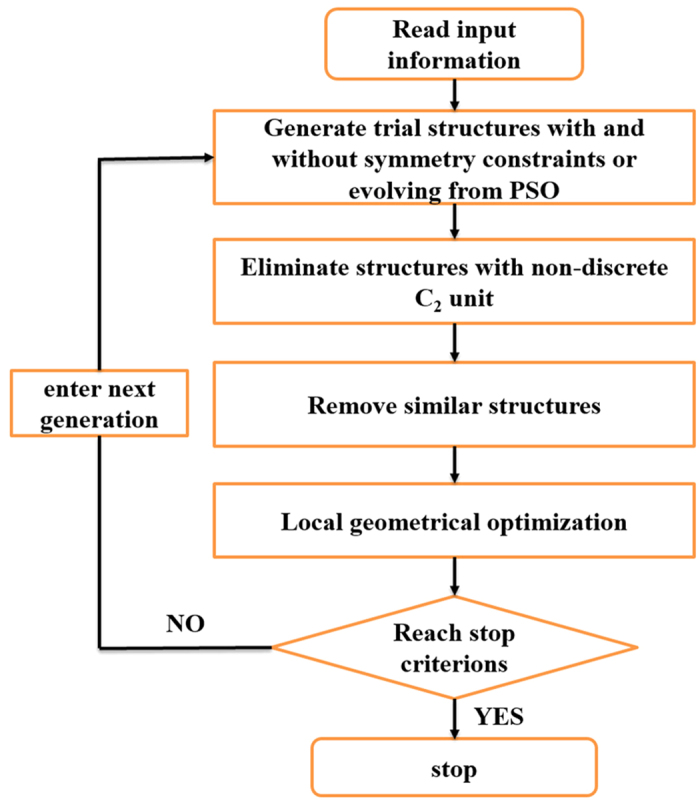
Flowchart of the dimer-based structure prediction code.

Due to the great success of PSO in predicting crystal structures, we have studied a dimer-based structure search program based on the PSO algorithm, where additional constraints and augmented algorithm are added. The main framework of our dimer-based structure search method is illustrated by the flowchart as shown in Fig. 5.

**Figure 6:**
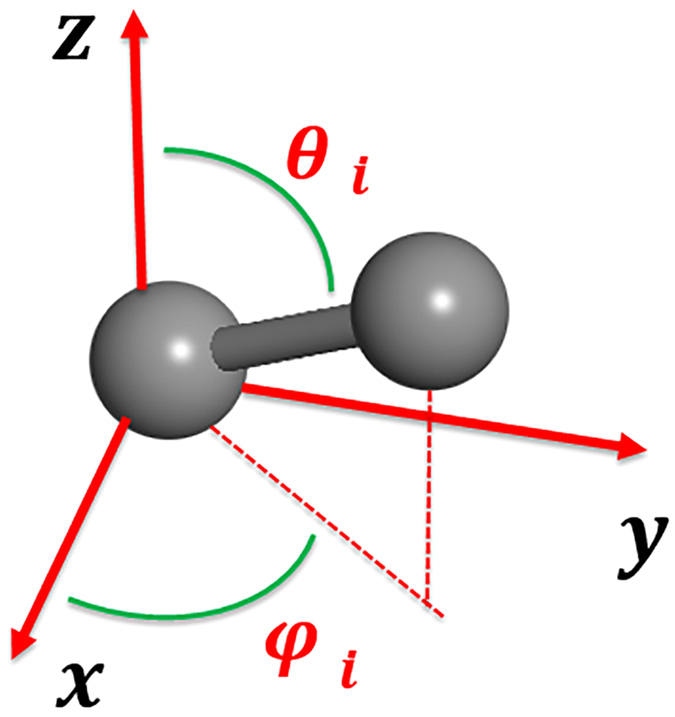
Coordinates (*x*_*i*_, *y*_*i*_, z_*i,*_*φ*_*i*_, *θ*_*i*_) for describing a dimer. (*x*_*i*_, *y*_*i*_, z_*i*_) are for the barycenter of C_2_ dimer, with (*φ*_*i*_, *θ*_*i*_) for its orientation.

Before outlining the details of the steps given in the flowchart, we will explain the treatment of the C_2_ dimer in our constrained search method. The requirements are that all carbon atoms exist in the form of a C_2_ dimer and each dimer must be separated from the others. A C_2_ dimer can be treated naturally as a “pseudo-atom”. However, general treatments for ordinary atoms are not sufficient for “pseudo atoms”, as an ordinary atom is treated as an isotropic sphere, while a C_2_ dimer is considered a pseudo atom exhibiting structural anisotropy along the molecular axis. Therefore, to fully represent a C_2_ dimer, extra indices are imperative. As shown in Fig. 6, besides the three coordinates, *x, y* and *z*, the dimeric orientation angles φ and θ are required. Therefore, in our augmented PSO algorithm, the coordinates needed are *r*_*i*_ = (*x*_*i*_, *y*_*i*_, *z*_*i*_, *φ*_*i*_, *θ*_*i*_), where *x*_*i*_, *y*_*i*_ and *z*_*i*_ are used to denote the barycenter, *φ*_*i*_ and *θ*_*i*_ are used to represent the orientation of the dimer. Besides, during the PSO-based structure searches, the bond length of C_2_ dimer is allowed to vary within the length of C-C single and triple bond. Accordingly, the PSO algorithm is modified to accommodate these changes.

**Figure 7:**
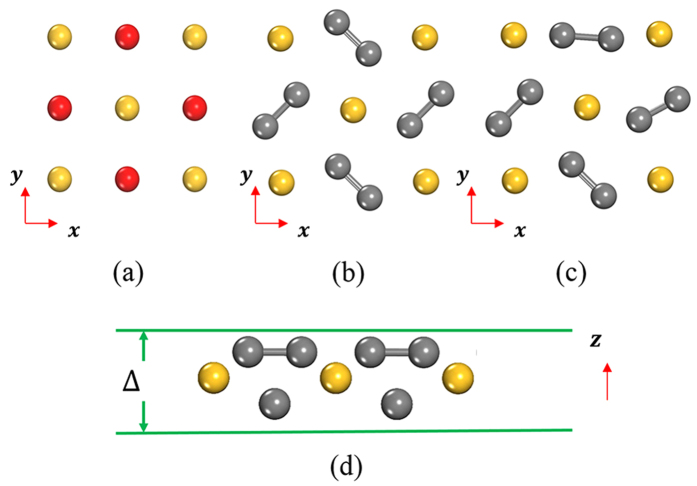
Illustration of procedure for generating dimer based structures. (**a**) Dimers are considered as pseudo-atoms (in red), and atoms and dimers are allocated to Wyckoff Positions allowed by symmetry. (**b**,**c**) for different orientations. (**d**) Buckled structures with a finite thickness of Δ in the z direction.

There are four main steps enumerated in the flowchart, some operative techniques are directly adapted from previous works^46^,^48^. First of all, most of the trial structures are partially randomly generated under symmetry restrictions^46^,^49^,^50^ or evolved *via* the PSO algorithm^48^,^51^. Fully randomly generated structures are also used. The reason is in 2D space there are 17 plane groups overall belonging to four different crystallographic systems (oblique, rectangular, square, and hexagonal); one of the groups is selected each time to create a new trial structure. The lattice shape of the new structure is determined by the selected space group and its area. Generally, the positions of the atoms or the barycenter of C_2_ dimer are allocated on Wyckoff positions for the chosen plane group. An extra step is needed to generate bond length and orientation for each dimer randomly or partially restricted, which is different from atom-based structure prediction methods. All these steps are sketched in Fig. 7. In order to predict 2D materials with finite thickness, the coordinate *z*_*i*_ of atoms and dimers are assigned randomly within a thickness Δ as shown in Fig. 7(d). However, because the number of 2D plane groups is finite, the generating method for a symmetry constrained structure is prone to produce similar structures. In this case totally randomly generated structures are needed to guarantee the diversity of structures.

Another source of the new trial structures is the PSO evolution which is a population-based algorithm. The population in our case is defined as a group of structures. For generation *t*, we define a population *P*_*t*_ and every structure in generation *t* is represented as a particle *P*_*i*_^*t*^. The evolution of structures is controlled by PSO algorithm:


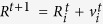


where *i* and *t* are indexes of particle (structure) and generation, 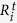 represents the structural information of a certain structure including the orientation of dimers and the coordinates of atoms, 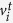 is the velocity or rate of structural change of certain structure *i*. The velocity of structure *i* is calculated as follows:


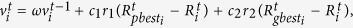


where *ω* is the inertia weight and controls the momentum of the particle, 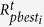 is the optimized structure of particle *i*, and 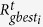 is the structure with the lowest energy obtained so far for generation *t, r*_1_ and *r*_2_ are two random numbers uniformly distributed in range [0, 1], *c*_1_ and *c*_2_ are set to be 2^47^,^52^. The definition of velocity fully reflects the idea of collective intelligence. The trajectory of each particle is biased, to a certain degree, toward the best structure of whole population and itself at the same time.

The three structure generation approaches work synergistically and serve different purposes during the structure search. The partially random structure generation with symmetry constraints help reduce the degree of freedom and narrow the search space. The fully random structure generation warrants the structural diversity, and the PSO-based structure evolution provides an effective way to explore the configuration space and locate the thermodynamically favorable structures.

It is important to note here that due to the nonequivalent coordinates *x*_*i*_, *y*_*i*_, *z*_*i*_, and *φ*_*i*_, *θ*_*i*_ in terms of their magnitudes and range, C_2_ dimers and ordinary atoms must be manipulated separately. In addition, the newly generated trial structures must satisfy the condition that all C_2_ dimers are disconnected from each other, thus the structures with dimer clustering are eliminated. Next, similarities between the different structures are examined. Each structure is characterized by a set of predetermined fingerprinting functions^48^,^52^, and the distance between two structures are calculated based on the difference of their fingerprinting functions. If a newly produced structure is similar to the previous one, the new structure is discarded. Finally, local structure optimization is performed to drive the structure to the local minimum on multi-dimensional energy space, which also provides physical and rational structures for future PSO evolution.

### First principles calculation

Combined with our bonding-restricted algorithm, first principles calculations within the framework of density functional theory (DFT) are performed for local geometry optimizations during structure search. Calculations are mainly performed by using the Vienna *Ab initio* Simulation Package (VASP)^53^. The Perdew-Burke-Ernzerhof (PBE) functional^54^ is used to incorporate the exchange-correlation interaction. The HSE06 hybrid functional^55^,^56^ is used for high accuracy of electronic structure calculations. The projector augmented wave (PAW)^57^ method is used to treat the interactions between ion cores and valance electrons. Plane waves are used to expand the valance electron (2*s*^2^2*p*^1^ for B, 2*s*^2^2*p*^2^ for C, 3*s*^2^3*p*^2^ for Si, and 3*d*^3^4*s*^1^ for Ti) wave functions. During the massive structure searching stage, in order to reduce the workload, plane waves with a kinetic energy cutoff of 350 eV and Monkhorst-Pack scheme with a sparse grid density (2π × 0.04 Å^−1^) are adopted. To compare the relative stability of different candidate structures, we used the same kinetic energy cutoff, k-point grid density and exchange-correlation functionals to perform geometry optimizations and total energy calculations. Plane waves with a kinetic energy cutoff of 450 eV and Monkhorst-Pack scheme^58^ with a grid density of 2π × 0.02 Å^−1^ are used to optimize the structures and calculate their electronic properties. The convergence criteria for total energy and forces are set to be 10^−4^ eV and 10^−3^ eV/Å, respectively. The supercell approach is used to calculate force constants. A vacuum space of 20 Å in the perpendicular direction is added to avoid the interaction between periodic images. Phonon properties are calculated using the finite displacement method as implemented in the Phonopy package^59^,^60^.

## Additional Information

**How to cite this article**: Zhang, C. *et al*. Bonding-restricted structure search for novel 2D materials with dispersed C_2_ dimers. *Sci. Rep.* **6**, 29531; doi: 10.1038/srep29531 (2016).

## Supplementary Material

Supplementary Information
