# Supplementary material for: Bonding-restricted structure search for novel 2D materials with dispersed C2 dimers
Source: Sci Rep. 2016 Jul 12;6:29531. doi: 10.1038/srep29531 (PMC4941692; doi:10.1038/srep29531)
Supplement: Supplementary Information [file srep29531-s1.doc]

Supplementary Information for

# **Bonding-restricted structure search for novel 2D materials with dispersed C_2_ dimers**

Cunzhi Zhang^1, 2^, Shunhong Zhang^2, 1^, and Qian Wang^2, 1, *^

^1^Department of Materials Science and Engineering, College of Engineering, Peking University, Beijing 100871, China

^2^Center for Applied Physics and Technology, Peking University, IFSA Collaborative Innovation Center, Key Laboratory of High Energy Density Physics Simulation, Ministry of Education, Beijing 100871, China

**Supplementary Figure S1. Dynamical stability of C_2_-based 2D SiC_2_ isomers. |** Phonon spectra of (a) penta-SiC_2_, (b) 456-SiC_2_ and (c) 46-SiC_2_.

The distinctive structural difference between different 2D SiC_2_ isomers (See Fig. 1) is the different orientations of C_2_ dimers. To obtain insight into the structural transformation between the different isomers, we investigate the energy barrier associated with the in-plane rotation of C_2_ dimers. For the sake of simplicity, we first use the ground state penta-SiC_2_ structure as an example and build a 2×2 supercell as the initial state, while final state is obtained by rotating one C_2_ dimer (marked in red) by 90°, as shown in Fig. S2 (a) and (b), respectively. We use the NEB method to determine the transformation path and the corresponding energy barrier. The calculated energy profile is presented in Fig. S2 (f), where one can see the energy barrier is around 3 eV. We then calculate the energy barrier between the penta-SiC_2_ and 456-SiC_2_ structures. The transformation from penta-SiC_2_ to 456-SiC_2_ requires the in-plane rotations of the two C_2_ dimers in a (√2×√2) R45° supercell, as shown in Fig. S2 (c-d). We decompose the transformation into two steps. First, by rotating the dimer marked in red in Fig. S2 (c) we yield an intermediate state shown in Fig. S2 (d). Second, the C_2_ dimer marked in blue in Fig. S2 (d) is rotated to reach the 456-SiC_2_ structure in Fig. S2 (e). The calculated kinetic barrier for the structure (c)-to-(d) and structure (d)-to-(e) transformations are about 3 eV and 4 eV, respectively, as plotted in Fig. S2 (g). The considerable kinetic barrier indicates that all these dimer-based 2D Si-C structures can exist as ground state or metastable configurations because the transformation requires substantial activation energy.”

**Supplementary Figure S2.** **Structural transformation between different 2D SiC_2_ structures. |** Sketches of C_2_ dimer rotation and associated energy profile. (a) A 2×2 supercell of penta-SiC_2_, and (b) a new supercell obtained by rotating the single C_2_ dimer marked in red in (a) by 90°. (c), (d) and (e) show the transformation from penta-SiC_2_ to 456-SiC_2_ structure *via* the in-plane rotations of the two C_2_ dimers marked in red and blue in a (√2×√2) R45° supercell of penta-SiC_2_. (f) Energy profile of single C_2_ dimer rotation corresponding to transformation from (a) to (b). (g) Energy profile corresponding to the transformation from (c) to (d), and finally to (e).

**Supplementary Figure S3.** **Structural details of C_2_-based 2D SiC_2_ isomers. |** The detailed geometrical configuration of Si and C in penta-SiC_2_ (a) and 46-SiC_2_ (b). Both the C and Si in 46-SiC_2_ subjects to greater distortion compared with that in penta-SiC_2_.

**Supplementary Figure S4.** **Dynamical stability of C_2_-based 2D B-C compounds. |** (a1) - (a3) Phonon spectra of three B_2_C_2_ isomers. (b) Phonon spectrum of BC_2_ sheet.

**Supplementary Figure S5.** **Dynamical stability of C_2_-based 2D Ti-C compounds. |** Phonon spectra of (a) TiC_2_ and (b) Ti_2_C_2_.

Here we briefly discussed the favorable conditions for the formation of Ti_2_C_2_ and TiC_2_ sheets proposed in our paper. We termed the ground state TiC_2_ sheet predicted in the present study (Fig. 4(a)) as TiC_2_-I, and the TiC_2_ structure suggested by Zhao et al. ^1^ as TiC_2_-II. In Fig. S3 of the paper by Zhao *et al.*, a phase diagram of Ti-C compounds is provided by using ab initio thermodynamics. Compared to the cohesive energy of Ti-C compounds in that diagram, TiC_2_-II is stable against decomposing into graphene and hcp-Ti, but is metastable when taking graphene and rocksalt-structured TiC as the reference state. TiC_2_-I is energetically more favorable than TiC_2_-II. Therefore, we propose that these C_2_ dimer-based titanium carbide sheets could be prepared in a carbon-rich environment by using graphene and fcc-Ti as the vapor source for surface growth.

**Supplementary References**

1 Zhao, T., Zhang, S., Guo, Y. & Wang, Q. T_i_C2: a new two-dimensional sheet beyond MXenes*. Nanosca*l**e** 8, 233-242, (2016).
